# Heparanase in cancer progression: Structure, substrate recognition and therapeutic potential

**DOI:** 10.3389/fchem.2022.926353

**Published:** 2022-09-09

**Authors:** Fengyan Yuan, Yiyuan Yang, Huiqin Zhou, Jing Quan, Chongyang Liu, Yi Wang, Yujing Zhang, Xing Yu

**Affiliations:** Key Laboratory of Model Animals and Stem Cell Biology of Hunan Province, School of Medicine, Hunan Normal University, Changsha, China

**Keywords:** glycosaminoglycan (GAG), heparanase, structure, substrate recognition, cancer

## Abstract

Heparanase, a member of the carbohydrate-active enzyme (CAZy) GH79 family, is an endo-β-glucuronidase capable of degrading the carbohydrate moiety of heparan sulphate proteoglycans, thus modulating and facilitating remodeling of the extracellular matrix. Heparanase activity is strongly associated with major human pathological complications, including but not limited to tumour progress, angiogenesis and inflammation, which make heparanase a valuable therapeutic target. Long-due crystallographic structures of human and bacterial heparanases have been recently determined. Though the overall architecture of human heparanase is generally comparable to that of bacterial glucuronidases, remarkable differences exist in their substrate recognition mode. Better understanding of regulatory mechanisms of heparanase in substrate recognition would provide novel insight into the anti-heparanase inhibitor development as well as potential clinical applications.

## Introduction

As a key component of the extracellular matrix (ECM), heparan sulfate proteoglycans (HSPGs) comprise of a transmembrane or secreted protein core to which one or more heparan sulfate (HS) chains are covalently attached (Iozzo, 2005; Iozzo and Schaefer, 2015). HSPGs are one of the most highly negatively charged biopolymers occurred naturally, collaborating with other ECM components to orchestrate the ECM remodeling and structural integrity (Belting, 2003; Lindahl and Kjellen, 2013). Significantly, HS chains of HSPGs act as a storage depot, providing binding sites for a wide variety of bioactive molecules, such as growth factors, chemokines, lipoproteins and enzymes, which enables HSPGs to play essential roles in regulation of numerous physiological and pathological activities (Varki et al., 2009; Iozzo and Schaefer, 2015).

Heparanase (HPSE; HPSE-1), a member of the glycoside hydrolase (GH) 79 family, has been defined as the only known endo-β-D-glucuronidase that catalyzes HS hydrolysis to date (Parish et al., 2001). Since the cloning and expression of HPSE in 1999, emerging evidence highlighted the involvement of HPSE in cancer progression, inflammation and angiogenesis (Fairbanks et al., 1999; Hulett et al., 1999; Kussie et al., 1999; Toyoshima and Nakajima, 1999; Vlodavsky et al., 1999). Of interest is that HPSE expression is elevated virtually in all major types of cancers, and this up-regulation is positively correlated with metastatic potential of tumor and poor prognosis, which makes HPSE a valuable therapeutic target. It has to be noted that another HPSE isoform, HPSE-2 that lacks enzymatic activity, was reported in 2000 (McKenzie et al., 2000). HPSE-2 appears not only to be able to inhibit HPSE activity but also regulate a multitude of signaling pathways that mediate cell differentiation, apoptosis and tumor vascularity, leading to tumor suppression.

Interpretation of the substrate specificity of HPSE has been complicated, partly if not all, by the nature of HS structural heterogeneity, which is derived from the extent of the sulfation, deacetylation and epimerisation in HS biosynthesis (Ringvall et al., 2000; Li et al., 2003; Pallerla et al., 2008). The structural features of HPSE also critically contribute to the plasticity in its substrate specificity, which is central to the proper biological function of HPSE. There are three members with available crystallographic structures to date in the GH79 family: Acidobacterium capsulatum β-glucuronidase (AcaGH79) (Michikawa et al., 2012), Burkholderia pseudomallei HPSE (BpHPSE) (Bohlmann et al., 2015) and human HPSE (hHPSE) (Wu et al., 2015) as well as its pro-form HPSE (hproHPSE) (Wu et al., 2017). The structure of the *exo*-acting AcaGH79 was characterized first, followed by the recent structural determination of *endo*-acting bpHPSE and hHPSE. Though hHPSE represents an overall similar folding to that of two bacterial GH79 members, structural variations within the substrate binding canyon fine-tune the distinct substrate specificities. Compelling evidence suggest that HPSE is a multifaceted protein participating in multiple biological processes, some excellent reviews are available pertaining to the engagement of HPSE in cancer progression, inflammation and angiogenesis (Fux et al., 2009b; Vlodavsky et al., 2012; Peterson and Liu, 2013; Pisano et al., 2014; Rivara et al., 2016; Masola et al., 2018; Mohan et al., 2019). In this minireview, we firstly provide an insight into the structure-based rationale of HPSE substrate recognition. Next, we briefly review the pro-tumorigenic effects of HPSE, which may highlight its therapeutic potential against cancer.

## Heparan sulfate proteoglycan

HSPGs consist of variable HS chains that covalently attach to core proteins depending on the context of source and growing conditions (Karamanos et al., 2018). HSPGs not only are present as crucial components of the ECM and basement membrane (Sertie et al., 2000; Arikawa-Hirasawa et al., 2001; Campos-Xavier et al., 2009), also are found in secreted vesicles regulating various biological activities after secretion (Zernichow et al., 2006), including membrane-bound syndecans, glypicans, betaglycan, neuropinlin and CD44v3, ECM components perlecan, agrin and collagen XVIII, and secreted serglycin. After the attachment of xylose to specific serine residues in core proteins of HSPGs, HS biosynthesis is commenced by synthesizing a linkage tetrasaccharide, glucuronic acid (GlcA)-galactose-galactose-xylose.

**FIGURE 1:**
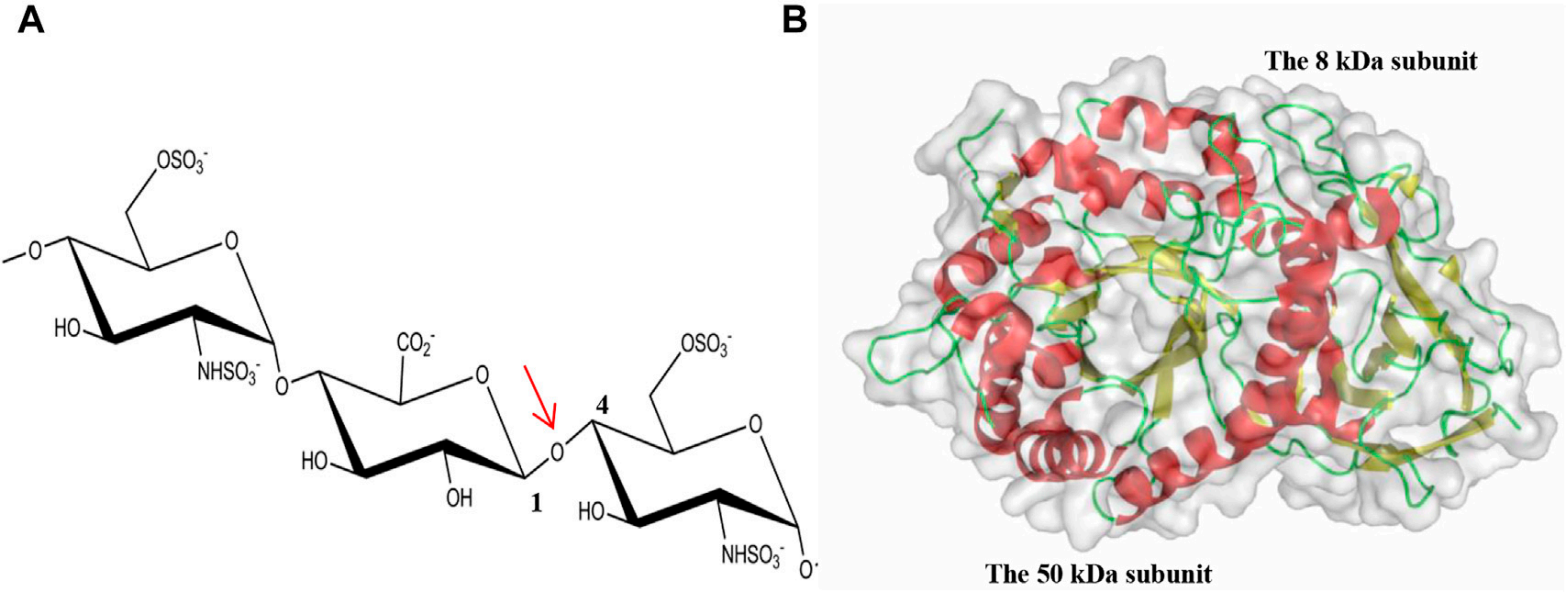
Cleavage of the glycosidic bond by HPSE. **(A)** The internal β(1,4)-linked glycosidic bond between GlcA and GlcNS is highlighted by an arrow in red; **(B)** The overall fold of hHPSE is illustrated in ribbon representation with α-helix in red, β-strand in yellow and loop in green.

Structurally, HS is a glycosaminoglycan (GAG) chain with potential modifications of sulfation, epimerization and deacetylation, comprising of a linear repeating disaccharide unit constituted by acetylated hexosamines (N-acetyl-glucosamine, GlcNAc or N-sulfo-glucosamine, GlcNS) and uronic acids (GlcA or its C5 epimer L-iduronic acid, IdoA) (Kjellen and Lindahl, 1991). Further O-sulfation can take place at O2 of the uronic acid (2-O sulfation) and O3 and O6 of the hexosamine (3-O and 6-O sulfation). Numerous combinations of the low sulfation and high sulfation domains along HS chains as well as the specific sulfation pattern within each domain complicate the recognition of HSPGs by HPSE. Early studies suggest that the minimum recognition backbone of HSPGs by HPSE is a trisaccharide, and the cleavage occurs at the internal β(1,4)-linked glycosidic bond between GlcA and GlcNS (Figure 1A) (Matsuno et al., 2002; Peterson and Liu, 2013). Further investigation revealed that HPSE cleavage of HSPG is dependent on sulfation types rather than a defined saccharide sequence, and the cleavage by HPSE is regulated by specific sulfation contexts around the cleavage site (Peterson and Liu, 2010).

The number of attached HS chains, together with the sulfation distribution along the HS chains, leads to high structural heterogeneity of HSPGs. In addition, modifications of HS occurred during its biosynthesis appear to be non-template, context-specific and in response to stimuli, therefore resulting in remarkable variations in HS chains (Armistead et al., 2011; Sarrazin et al., 2011; Shi et al., 2011). Of relevance is that the structural heterogeneity of HS facilitates its capability of accommodating a variety of binding partners, which is essential to the diverse biological roles of HSPGs upon HPSE breakdown, leading to activation of downstream signal cascades and promotion of cell proliferation, tumor cell dissemination, inflammation and angiogenesis (Bernfield et al., 1999; Elkin et al., 2001; Iozzo and San Antonio, 2001; Sasaki et al., 2004; Barash et al., 2010b; Goodall et al., 2014). Previous studies have demonstrated that hHPSE is able to act in either a consecutive or a gapped cleavage mode depending on the saccharide sequences released from its initial cleavage (Peterson and Liu, 2013), which allows the efficient release of distinct bioactive molecules from regions with different sulfation patterns along HS chains.

## HPSE

### Overview

The gene coding for HPSE consisting of 14 exons and 13 introns is located on chromosome 4q21.3 and expressed as two mRNAs (5 and 1.7 kb) by alternative splicing containing the same open reading frame (Dong et al., 2000). HPSE is initially synthesized as a preproenzyme of 68 kDa containing a signal sequence spanning Met1–Ala35, which is then processed into a proHPSE form after cleaving the signal sequence by signal peptidase. Lysosomal activation by cathepsin L excises a linker domain of Ser110–Gln157, giving rise to the mature HPSE as a non-covalent heterodimer containing an N-terminal 8 kDa (Gln36-Glu109) and a C-terminal 50 kDa (Lys158-Ile543) subunits.

β-glucuronidases are categorized into three GH families, GH1, GH2, and GH79, on the basis of their amino acid sequences (Henrissat and Davies, 1997; Cantarel et al., 2009). There are four characterized β-glucuronidase members in the GH79 family, including heparanase (EC 3.2.1.166), baicalin-β-D-glucuronidase (EC 3.2.1.167), 4-O-methyl-β-glucuronidase, and β-glucuronidase (Sasaki et al., 2000; Parish et al., 2001; Eudes et al., 2008; Konishi et al., 2008). Though both GH2 and GH79 belong to the GH-A clan, the GH79 family is composed of enzymes of both *endo*-acting HPSE and *exo*-acting β-glucuronidase, which contrasts that the GH2 family only consists of *exo*-acting β-glucuronidase. Folding prediction as well as multiple sequence alignment has predicted HPSE being a member of GH-A clan, proposing An (β/α)_8_-TIM barrel as the key folding feature of HPSE (Nardella et al., 2004). This was confirmed after the recent determination of the long-anticipated hHPSE and hproHPSE structures.

### HPSE structure and substrate recognition

The structure of apo hHPSE consists of a heterodimer formed by the 8-kDa subunit (residues Gln36–Glu109) and the 50-kDa subunit (Lys159–Ile543) (Wu et al., 2015), with the domain architecture comprising a catalytic (β/α)8-TIM barrel domain flanked by a β-sandwich domain (Figure 1B). Both the 8-kDa subunit and the 50-kDa subunit contribute to the formation of the catalytic (β/α)_8_-TIM barrel and the β-sandwich domain. Though the β-sandwich domain was reported to facilitate secretion and activation, cellular trafficking, enzymatic and nonenzymatic activities of HPSE, its function demands to be further characterized (Simizu et al., 2007; Lai et al., 2008; Fux et al., 2009a). In addition, there are six putative N-glycosylation sites identified in the 50-kDa subunit of hHPSE. After the deglycosylation treatment of Endo-H during the protein preparation, N-linked GlcNAc residues were visible in the apo hHPSE structure at Asn162, Asn200, Asn217, Asn238 and Asn459, respectively (Wu et al., 2015). Intriguingly, glycosylation regulates HPSE secretion and endoplasmic reticulum-to-Golgi transport, but it is not required for enzymatic activity of HPSE (Simizu et al., 2004).

**FIGURE 2:**
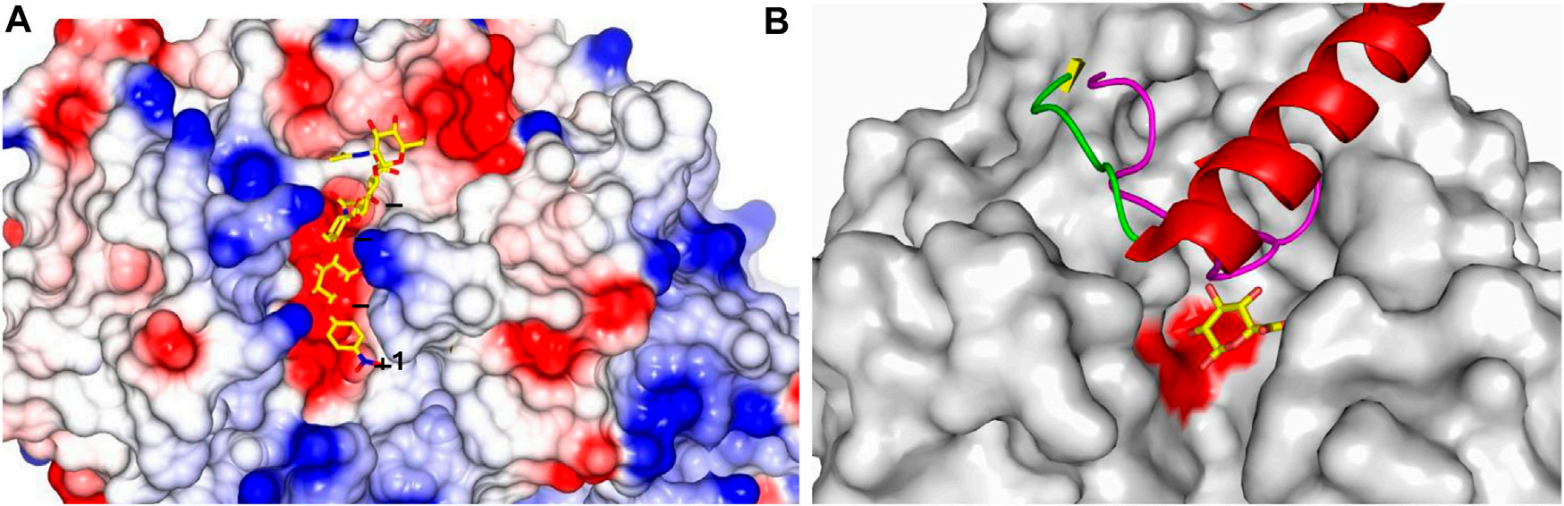
HPSE substrate binding site and structural superimposition. **(A)** hHPSE (electrostatic surface) in complex with a bound tetrasaccharide (our unpublished work) in stick presentation (carbon in yellow, nitrogen in blue and oxygen in red) spanning through binding subsites +1, −1, −2, −3 (Davies et al., 1997); **(B)** BpHPSE is illustrated in surface representation with two conserved catalytic glutamate highlighted in red; the loop that forms part of the substrate-binding pocket in AcaGH79 is colored in magenta; the 6-kDa linker of hproHPSE is illustrated in ribbon representation with α-helix in red, β-strand in yellow and loop in green; the bound GlcA are colored with carbon in yellow and oxygen in red.

A binding groove of approximately 10 Å in the catalytic (β/α)_8_-TIM barrel domain was recognized in the hHPSE structure. This binding groove contains residues Glu343 and Glu225, which are conserved in the GH79 family and have been previously identified as the catalytic nucleophile and acid-base pair of HPSE, suggesting that the HS-binding site is contained within this groove (Hulett et al., 2000). As shown in Figure 2A, the HPSE binding canyon is lined by side chains of basic residues, which correlates well with the negatively charged nature of HS substrates. Of interest is that the orientation of two subunits in the solved hHPSE structure implicates that the excised Ser110–Gln157 linker of the proHPSE could locate very close in space to the HS binding groove, which would physically clash the HS substrate. This is consistent with the reported hproHPSE structure in 2017, showing the restricted access to the active site cleft for oligosaccharide HS substrates due to the presence of the 6-kDa linker loop (Figure 2B) (Wu et al., 2017).

Structures of hHPSE in complex with HS analogues provides a structural rationale, clearly demonstrating that hHPSE recognizes a trisaccharide spanning the −2, −1 and +1 subsites, with the identical binding of GlcA at the −1 subsite in all bound HS analogues (Figure 2A). This conserved binding of GlcA is also observed in the GH79 bacterial members, suggesting a key GH79 structural motif that has been fine tuned to recognize GlcA (Michikawa et al., 2012; Wu et al., 2015). Significantly, N-sulfate at the −2 subsite and 6O-sulfate at the +1 subsite appear to be the main determinants for recognition of HS analogues due to their direct engagement in the hydrogen-bonding interactions with HPSE. 6O-sulfate at the −2 subsite and N-sulfate at the +1 subsite also contribute to the anchorage of HS substrates through electrostatic interactions to basic residues lining the active site cleft. Overall, structural information gained from the complexes of hHPSE with its HS substrate analogs is consistent with the findings of previous studies that HS sulfation patterns are essential for hHPSE enzymatic activity. Furthermore, sulfation contexts of HS substrates appear to act as a molecular signal that guides the precise cleavage of designated glycan sites. In addition, sulfate groups on the −2 and +1 moieties are implicated to aid hHPSE to unwind the substrate HS helix for a better access of the catalytic residues to facilitate the cleavage of the glycosidic bond.

### Structure-based rationale for the *exo*- and *endo*-acting modes of GH79 β-glucuronidases

When compared in the sequence alignment, a loop of 40 amino acids (Gly78–Thr117), which forms part of the *exo*-acting substrate-binding pocket identified in the AcaGH79 structure, corresponds to its counterpart that is substantially reduced in size of *endo*-acting BpHPSE (24 amino acids, Gly67–Pro90). Structurally, this shorter loop of BpHPSE allows the transition of the binding pocket into an open-end binding groove capable of accommodating elongated HS chains, which hereby provides a well-explained structure-based rationale of the discrepancy in acting modes of the substrate cleavage between the *exo*-AcaGH79 and *endo*-BpHPSE enzymes (Figure 2B).

The overall folding of hHPSE is comparable to that of two characterized bacterial GH79 members (Michikawa et al., 2012; Bohlmann et al., 2015), with Cα r.m.s. differences of 2.35 Å and 2.59 Å for AcaGH79 and BpHPSE, respectively. In particular, the 6 kDa linker peptide of hproHPSE that is proteolytically cleaved to enable the activation of hHPSE also corresponds to the AcaGH79 loop. Intriguingly, structural observations revealed that the physical presence of the hproHPSE linker peptide create a binding pocket on the protein surface containing those two highly conserved glutamate, resembling some structural characteristics of the *exo*-acting active site of AcGH79 (Wu et al., 2017). Detailed data indicate that this hproHPSE pocket is not involved in the HS interactions and GlcA occupation of the proHPSE pocket does not inhibit HPSE maturation, suggesting extra subsite interactions may be required for anchoring single GlcA molecules to proHPSE. Further investigations are thus required to determine whether this proHPSE pocket possesses any substrate specificity.

Whereas both hHPSE and BpHPSE demonstrate the *endo*-acting mode of substrate cleavage, differences exist between their substrates recognition. BpHPSE has preference for cleaving HS-containing GlcNAc residues (low sulfation), contrasting that GlcNS is preferably recognized by hHPSE. Moreover, sequence alignment of several eukaryotic HPSEs with BpHPSE and AcaGH79 revealed a remarkable conservation of key residues for accommodating GlcA at the −1 subsite of hHPSE, demonstrated by absolute conservation of residue Asn224, Glu225, Glu343, Gly350 and Tyr391 and evolutionary conservation of residue 62 (Asp of hHPSE vs Glu of BpHPSE and AcaGH79), 97 (Thr of hHPSE vs. Asn of BpHPSE and AcaGH79), 349 (Gly of hHPSE and BpHPSE vs Gln of AcaGH79), whilst residues at the −2 and +1 subsites show much poorer conservation in the BpHPSE and AcaGH79, thus providing a structure-based rationale for distinct substrate specificity amongst those GH79 enzymes (Wu et al., 2015).

### Transport and function of HPSE in nucleus and extracellular

It is generally known that mature HPSE is the only known endo-β-D-glucuronidase in mammals, which can cleave the HS chain of HSPG to release growth factors, chemokines, lipoproteins and enzymes, play a role in promoting tumors outside the cell. Many studies have shown that the function of HPSE is regulated by histones. Histones mainly exist in the nucleus and extracellular regions and secreted proteins. Histones (H1, H2A, H2B, H3, and H4) contain a large number of basic R-based amino acids, which are positively charged in aqueous solutions. While HSPG as one of the most negatively charged biopolymers, HSPG and histones bind to the GAG chain of HSPG through charge interaction to play a regulatory role. For example, extracellular histone H4 induces HS degradation by activating HPSE in chlorine (Cl2)-induced acute respiratory distress syndrome (ARDS). Knockdown of HPSE by RNAi demonstrated that histone h4-induced HS degradation requires HPSE and is dependent on the enzymatic activity of HPSE (Zhang et al., 2022). In cells expressing high levels of HPSE, reduction of nuclear syndecan-1 results in increased histone acetyltransferase (HAT) activity, which stimulates protein transcription and transcriptional upregulation of multiple genes that drive aggressive tumor phenotypes (Purushothaman et al., 2011). In lymphangiosarcoma (SS), histone deacetylase inhibitors (HDACi) upregulate HPSE by inducing the expression of the positive regulator EGR1 and inhibit the negative regulation of p53 by acetylation. By co-treatment with MEK inhibitor (trametinib) or HPSE inhibitor (SST0001/rooneparstat), blocking HDACi-induced erk-egr1-HPSE pathway enhanced antiproliferative and proapoptotic effects (Lanzi et al., 2021).

With the deepening of research, it was found that HPSE can inhibit tumor after entering the nucleus. Human HPSE sequences contain two potential nuclear localization signals (residues 271-277; PRRKTAK and residues 427-430; KRRK), which mediate nuclear localization of enzymes. Secondly, HPSE nuclear translocation can be promoted by its heparin binding domain, using HS as its carrier (Nadav et al., 2002). Yang et al. (2015) using atomic force microscopy and co-precipitation methods, found a direct molecular interaction between HPSE and DNA driven by charge, indicating that HPSE has dual functions in malignant melanoma, with primary extracellular activity and tumor-suppressive nuclear effect. In type 1 diabetes, heparin and HS can be transported to the nucleus and directly or indirectly affect gene transcription. Based on Chip-on-chip studies, heparin interacts with promoters and transcription regions of hundreds of genes and micro-RNAs in activated Jurkat T cells and upregulates transcription at the molecular level. Nuclear HPSE appears to regulate methylation of histone 3 lysine 4 (H3K4) by influencing demethylase recruitment of transcription-active genes (Parish et al., 2013).

### HPSE in cancer and its therapeutic potential

**FIGURE 3:**
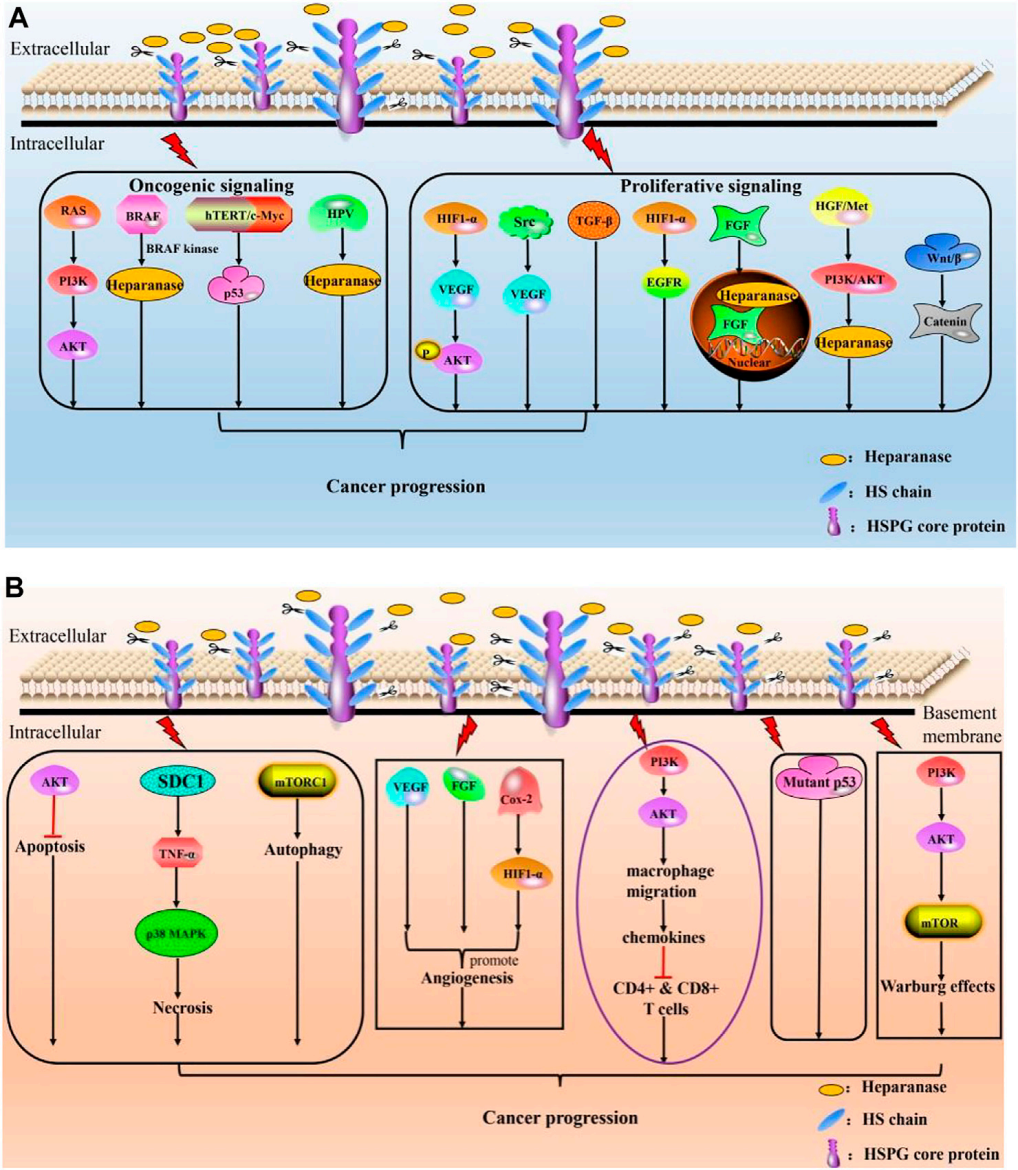
Roles of HPSE in cancer progression. **(A)** HPSE modulates cancer progression by mediating oncogenic signaling and proliferative signaling; **(B)** HPSE promotes cancer by resisting cell death, initiating angiogenesis, contributing to anti-immunity failure, circumventing growth inhibition and reprogramming energy metabolism.

As aforementioned, quite a few excellent reviews are available pertaining to activities of HPSE in different physiological and pathological contexts, we thus briefly summarize the pleiotropic actions of HPSE herein and will not go into detailed discussion (Figure 3). Function of HPSE is strongly associated with major human pathological complications, evidenced by that various literatures have linked overexpression of HPSE to enhanced tumor growth, metastasis and poor prognosis. Further, silencing of HPSE or treatment of tumor with compounds that block HPSE activity is shown to remarkably attenuate tumor progression. Therefore, targeting HPSE is considered as a promising therapeutic strategy for cancer treatment. Several classes of inhibitors have been developed, ranging from nucleic acid-based inhibitor, vaccines, MicroRNAs, anti-HPSE monoclonal antibodies, poly-sulfated saccharides to small-molecule inhibitors (Rivara et al., 2016). Though MicroRNAs and anti-HPSE antibodies are demonstrated to have high specificity, none of those so-called biological drugs, such as vaccines, antibodies, and antisense RNAs, have ever passed the clinical trials. Further, small molecule drugs also failed to enter clinical studies.

To the best of our knowledge, only few polysaccharide-based candidates synthesized by either semi-synthetic or total synthesis methods are currently clinical tested by competitively targeting the substrate binding site of HPSE. Irrespective of distinct mechanisms of action, those polysaccharide-based inhibitors, such as PI-88, M-402, PG545, and SST0001, appear to be the most promising anti-tumor agents due to their specificity and reasonable druggability. Significantly, the development of HPSE inhibitors still exist several drawbacks, among of which are structural uncertainty, per-sulfation, *in vivo* instability, poor bioavailability and apparent side effects (Rosenthal et al., 2002; Levidiotis et al., 2004; Hossain et al., 2010). As a result, novel strategies are emerging to develop HPSE inhibitors with higher specificity and greater selectivity (Sletten et al., 2017). Intriguingly, recent findings disclose that HPSE-2, a close homolog of HPSE but lacks enzymatic activity, can regulate antitumor mechanisms. However, this theme is not the main focus of this minireview, therefore it will not be further discussed.

## Conclusions and future perspective

Intensive studies have demonstrated that increased levels of HPSE expression are strongly associated with a multiplicity of hematological and solid malignancies. To this end, HPSE has become a promising target for fighting cancer. The therapeutic potential of HS mimetics, due to their ability to bind and modulate the function of HPSE, has therefore been exploited. Although several HS mimetics have advanced into clinical trials, unforeseen adverse effects are documented due to the heterogeneous nature and nonspecific or pleiotropic effects of those HS mimetics (Kudchadkar et al., 2008; Zhou et al., 2011).

Further, HPSE is a multifaceted protein having both enzymatic and non-enzymatic activities. To the best of our knowledge, all HPSE inhibitors under development are predominately targeting on the enzymatic inhibition of HPSE. Therefore, one main question raised in the development of anti-HPSE inhibitors is whether the enzymatic activity of HPSE is the critical determinant of its pro-tumor and pro-metastasis effects, given the fact that the T5 splice variant of HPSE lacking its enzymatic activity exerts roles in promotion of tumor progress (Barash et al., 2010a; Barash et al., 2019). Intensive studies are thus required to further explore non-enzymatic activities of HPSE attributed to its physiological and pathological function.

Recent determination of crystallographic structures of human and bacterial HPSE could offer an improved understanding of mechanisms of action of HPSE at the atomic level, which will greatly aid the design of HPSE inhibitors. Given the anti-tumor action of HS mimetics appears to be context-dependent and in response to external stimuli, it is advisable to develop HS mimetics as inhibitors in a system where appropriate malignancies and patient population are rationally selected for clinical trials. In addition, HS mimetics are characterized by good safety and tolerability profiles, which make them highly suitable for inclusion in combined therapies with other drugs to enhance anti-tumor efficacy of conventional treatments.
